# Immobilization of Bone Morphogenetic Protein on DOPA- or Dopamine-Treated Titanium Surfaces to Enhance Osseointegration

**DOI:** 10.1155/2013/265980

**Published:** 2013-12-28

**Authors:** Jeonghwa Kang, Seiichi Tada, Takashi Kitajima, Tae Il Son, Toshiro Aigaki, Yoshihiro Ito

**Affiliations:** ^1^Nano Medical Engineering Laboratory, RIKEN, 2-1 Hirosawa, Wako-shi, Saitama 351-0198, Japan; ^2^Department of Biological Science, Tokyo Metropolitan University, Minami-Osawa, Tokyo 192-0938, Japan; ^3^Department of Biotechnology and Bio-Environmental Technology (BET) Research Institute, Chung-Ang University, 40-1 San, Nae-Ri, Daeduck-myun, Ansung-si, Kyungki-do 456-756, Republic of Korea

## Abstract

Titanium was treated with 3,4-dihydroxy-L-phenylalanine (DOPA) or dopamine to immobilize bone morphogenetic protein-2 (BMP2), a biomolecule. DOPA and dopamine solutions turned into suspensions, and precipitates were produced at high pH. Both treatments produced a brown surface on titanium that was thicker at high pH than low pH. Dopamine produced a thicker layer than DOPA. The hydrophobicity of the surfaces increased after treatment with dopamine independent of pH. Furthermore, there were more amino groups in the layers formed at pH 8.5 than pH 4.5 in both treatments. Dopamine treatment produced more amino groups in the layer than DOPA. BMP2 was immobilized on the treated surfaces via a coupling reaction using carbodiimide. More BMP2 was immobilized on surfaces treated at pH 8.5 than pH 4.5 in both treatments. The immobilized BMP induced specific signal transduction and alkali phosphatase, a differentiation marker. Thus, the present study demonstrates that titanium treated with DOPA or dopamine can become bioactive via the surface immobilization of BMP2, which induces specific signal transduction.

## 1. Introduction

Biomedical engineering has the potential to improve the quality of human life. Chemical modification of biological signaling molecules such as cell growth factors on implants is important in clinical therapeutics. Titanium is a biocompatible implant material but does not have specific bio-functionality. The adsorption of plasma proteins onto titanium surfaces plays an essential role in implant integration. The bioactivation of implants requires the functionalization of an implant surface with signaling molecules [1–3].

The formation of new bone is required for successful outcomes in bone fracture repair and dental implants. Efficient bone formation depends on the recruitment of osteoblast precursors to the site followed by osteoblast maturation, matrix deposition, and mineralization [4, 5]. Bone morphogenetic protein-2 (BMP2) is a signaling protein known to play important roles in the bone healing process and enhancing therapeutic efficacy [6, 7]. Therefore, coating or immobilizing BMP2 onto organic or inorganic surfaces is reported to enhance the osseointegration of materials [8–16].

Some researchers report physically coating titanium with BMP [17–19]. In addition, Kashiwagi et al. [20] prepared titanium-binding BMP using their selective titanium-binding peptide. On the other hand, in order to create stable covalent immobilization, Puleo et al. [21] performed plasma polymerization of allylamine on a titanium surface. Meanwhile, others prepared chitosan, dextran, or polymer layers on titanium to covalently immobilize BMP [22–25].

However, the covalent modification method of inorganic surfaces is limited, although there are some specific methodologies such as silane coupling. Therefore, Lee et al. [26, 27] devised a new convenient and universal method. Underwater adhesive proteins containing 3,4-dihydroxy-l-phenylalanine (DOPA) from mussel protein play important roles in adhesion to various materials including polymers, metals, and ceramics. Therefore, Lee et al. hypothesized that the coexistence of catechol (i.e., DOPA) and amine (i.e., lysine) groups is crucial for achieving adhesion to a wide variety of materials. They consequently identified dopamine as a small-molecule compound that contains both functionalities and found that it is useful for the surface modification of various materials [26, 27]. Material surfaces were treated with dopamine to immobilize biological molecules including growth factors [28–34]. This dopamine treatment resulted in “polydopamine” or “melanin-like” films produced through the oxidation of dopamine or other catecholamines such as norepinephrine. Thus, this represents a very convenient and universal method for adding an organic layer to various materials including polymers, metals, and ceramics.

Meanwhile, Lai et al. [35] utilized this dopamine treatment method to conjugate BMP on titanium for the first time; the covalent conjugation was performed under alkaline conditions as suggested by Lee et al. [28]. The surface functionalization of TiO_2_ nanotubes with BMP2 was beneficial for mesenchymal stem cell proliferation and differentiation. Their approach hints at potential applications in enhanced bone osseointegration stemming from the development of titanium-based implants.

We previously found that dopamine-treated surfaces contain amino groups that can be utilized for protein immobilization [33]. Therefore, in this study, we covalently immobilized BMP2 on dopamine-treated titanium surfaces using the amino groups. In addition to dopamine, DOPA was used for surface treatment as a link between titanium and BMP2, and the effect of BMP2 immobilization on titanium surfaces was investigated.

## 2. Materials and Methods

### 2.1. Materials

DOPA was purchased from Sigma (St. Louis, MO, USA). 3,4-Dihydroxyphenethylamine hydrochloride (dopamine) and *N*-hydroxysuccinimide (NHS) were purchased from Wako Pure Chemical Industries (Osaka, Japan). 1-Ethyl-3-(3-dimethylaminopropyl) carbodiimide hydrochloride (water-soluble carbodiimide (WSC)) was obtained from Dojindo (Kumamoto, Japan).

A glass plate (diameter, 15 mm; thickness, 1 mm) was coated by vacuum deposition with titanium (400 nm thick (±25%)) by Osaka Vacuum Industries Co. (Osaka, Japan) as previously reported [33].

Recombinant human BMP2 was purchased from R&D Systems Inc. (Minneapolis, MN, USA). Polyclonal anti-human BMP2 antibody was purchased from Abcam (Cambridge, UK). Horseradish peroxidase (HRP)-conjugated secondary antibody was obtained from Zymed (Carlsbad, CA, USA). Block Ace Powder was obtained from DS Pharma Biomedical (Sapporo, Japan).

### 2.2. Solution Measurement

Dopamine solution (2 mg/mL) was prepared in 10 mM Tris-buffer (adjusted to various pH values). After reacting at room temperature for 24 h, ultraviolet (UV) measurement was performed. Detection was carried out on the basis of using the absorbance at 500 nm. UV measurement was performed using a JASCO V-550 (Tokyo, Japan).

### 2.3. Surface Treatment

The surfaces of the plates were washed in hexane solution, cleaned with 6 M hydrogen chloride for 10 min, rinsed twice with triple-distilled water, dried in a vacuum oven for 24 h, and cleaned photochemically using an excimer UV lamp (USHIO Inc., Tokyo, Japan) for 10 min before incubation in dopamine solution; this method was applied to completely remove C–C bonds and avoid the subsequent decomposition of organic molecules. The complete removal of organic material was confirmed by the observed decrease in the water contact angle.

Next, DOPA or dopamine treatment was performed. The cleaned plates were placed in a flask containing 2 mg/mL dopamine or DOPA solution in water (pH 4.5) or 10 mM Tris-buffer (adjusted to pH 8.5). The reaction was performed at room temperature for 24 h. The treated TiO_2_ was rinsed in fresh water and dried in a clean vacuum oven at room temperature for 24 h.

To immobilize BMP2 on the surfaces of the plates, BMP2 solution was mixed with an aqueous solution of 50 mM WSC and 20 mM NHS. The treated plates were immersed in the mixed solution for 48 h at 4°C. After incubation, the plates were washed 3 times with phosphate-buffered saline (PBS).

### 2.4. Surface Analysis

The static water contact angles of the sample surfaces were measured at 25°C in air by a contact-angle meter (Kyowa Interface Science Co., Tokyo, Japan) based on the sessile drop method. All contact angles were determined by averaging 10 different point values measured on each dopamine-treated surface.

The thickness of the polymer was measured by an ellipsometer M-2000DI (JA Woollam Company, NE, USA) from 195 to 1,500 nm at 3 angles: 65°, 70°, and 75°. The surface roughness was analyzed by a New View 5032 apparatus (Zygo Co., Middlefield, CT, USA).

Fluorescein isothiocyanate (FITC) was used to determine the amount of amino groups on a surface. FITC solution (100 *μ*L, 10 mg/mL) in dimethylsulfoxide was mixed with 1 mL 0.1 M sodium bicarbonate solution (pH 9.0). The sample plate modified with DOPA or dopamine was incubated in the solution at room temperature for 1 h and subsequently rinsed 10 times with PBS. FITC was quantified by an AxioVision instrument (Zeiss, Oberkochen, Germany) with a Cool SNAP HQ camera (Photometrics, Tokyo, Japan).

Immobilized BMP2 was detected using an anti-BMP2 antibody. The plate was rinsed with PBS-Tween (PBS-T) (0.1%) and blocked by incubation in an aqueous solution of 1% nonfat milk for 30 min. The plate was subsequently incubated with an anti-BMP2 antibody (1 : 2,000 dilution) overnight at 4°C and washed 3 times with PBS-T (0.1%) before being incubated with an HRP-conjugated secondary antibody (1 : 10,000 dilution) for 1 h at room temperature. After washing 3 times with PBS-T (0.1%), a chemiluminescence reaction was performed using an ECL Plus Western Blotting Detection System (GE Healthcare, Fairfield, CT, USA) and was observed by Light-Capture (ATTO, Tokyo, Japan).

### 2.5. Cell Culture

BRE-Luc C2C12 cells, which have a luciferase reporter gene with a BMP2-specific enhancer derived from inhibitor of differentiation (Id)1 promoter, were cultured in DMEM (Sigma, St. Louis, MO, USA) supplemented with 5% fetal bovine serum (Moregate Inc., Hamilton, Waikato, New Zealand) and 1% penicillin-streptomycin (Wako Pure Chemical Industries, Osaka, Japan) at 37°C in 95% humidified air/5% CO_2_. The cells were then washed with 5 mL PBS and harvested using a 0.25% trypsin solution containing 1 mM EDTA (Wako Pure Chemical Industries, Osaka, Japan) for 3 min at 37°C. Finally, the recovered cells were suspended in medium for the subsequent* in vitro* examination.

To monitor BMP signaling, the cell suspension was added to 24-well tissue culture polystyrene plates (0.5 mL/well, 1 × 10^5^ cells/mL) containing the samples, which were previously washed with sterilized PBS. After the cells were cultured in a 5% CO_2_ atmosphere at 37°C for 48 h, they were washed with PBS and disrupted with lysis reagent (Promega, Madison, WI, USA). The luciferase activity in the lysate was measured using a luciferase assay reagent kit (Promega, Madison, WI, USA) with a Mithras LB940 luminescence plate reader (Berthold Technologies, Bad Wildbad, Germany). The observed activity was normalized to the protein content in the cell lysate, which was determined using a BCA protein assay kit (Pierce, Rockford, IL, USA).

As a marker of osteogenetic differentiation, alkaline phosphatase activity was measured as previously reported [36]. C2C12 cells suspension was added to 24-well tissue culture polystyrene plates (0.5 mL/well, 5 × 10^3^ cells/mL) containing the samples, which were previously washed with sterilized PBS. The cells were cultured in a 5% CO_2_ atmosphere at 37°C for 10 days (changing the media every 2 days), washed with Tris-buffered saline, and disrupted with Tris-buffered saline containing 0.2% Triton X-100. The alkaline phosphatase activity was measured using the fluorescent substrate, 4-methylumbelliferyl phosphate (Sigma, St. Louis, MO, USA), with a Mithras LB940 luminescence plate reader. The observed activity was normalized to the protein content in the cell lysate, which was determined using a BCA protein assay kit.

### 2.6. Statistical Analysis

Statistical analyses were performed using Student's *t*-test for paired samples and analysis of variance for multiple samples.

## 3. Results and Discussion

### 3.1. DOPA and Dopamine Treatment

**Figure 1:**
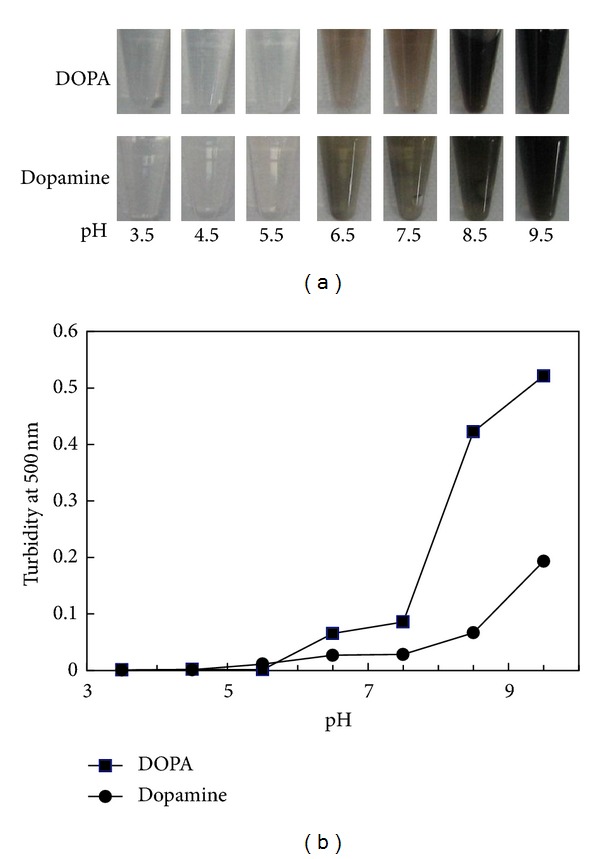
Photograph (a) and turbidity (b) of DOPA and dopamine solutions at different pH values. The turbidity data of dopamine solution are from Kang et al. [33].

The properties of DOPA and dopamine solution were investigated on the basis of turbidity (Figure 1). Both solutions were transparent at low pH even after 24 h and turned turbid and brown at high pH. After 24 h, some precipitate was found in dopamine solution at high pH. Although the turbidity change of the DOPA solution appeared to be greater than that of dopamine, the lower turbidity was due to the precipitation of aggregated dopamine. Therefore, dopamine was considered more reactive than on DOPA.

**Figure 2:**
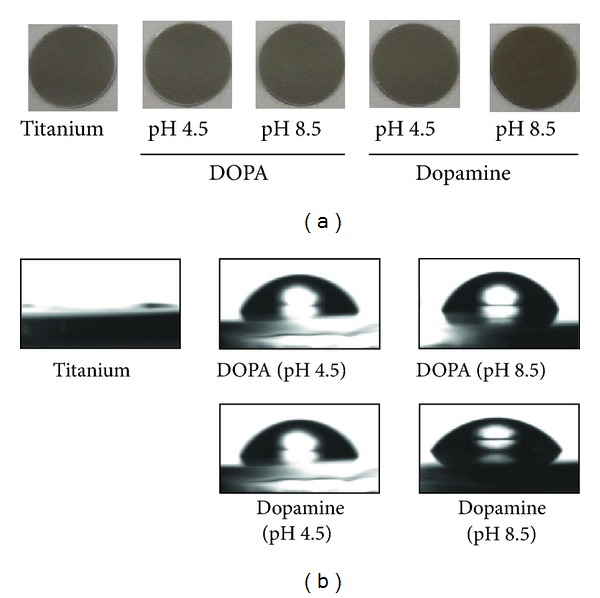
Images (a) and water contact angle (b) of DOPA- and dopamine-treated titanium.

When titanium-coated glass was treated with either DOPA or dopamine, the surface turned brown at pH 8.5 (Figure 2). The brown color was denser on dopamine-treated surfaces than DOPA-treated surfaces. In contrast, no significant color change was observed when the surfaces were treated at pH 4.5. The color change coincided with the thickness. The layer formed by DOPA was thinner than that formed by dopamine (Table 1). In the case of dopamine, the formed layer at pH 8.5 was about 28 times thicker than that formed at pH 4.5 (Table 1); in the case of DOPA, the surface was less than 5 times thicker.

However, the assessment of surface hydrophilicity on the basis of contact angle measurements revealed that the water contact angle of surfaces increased with DOPA or dopamine treatment even at pH 4.5; the contact angles on the surface were almost the same with treatment at pH 4.5 and pH 8.5 (Table 1). This indicates that the titanium surfaces were fully covered by DOPA or dopamine at pH 4.5 as described previously [33]. Therefore, the present results indicate the effect of dopamine is stronger than that of DOPA. The carboxyl group in DOPA is specifically considered to reduce the reactivity.

**Figure 3:**
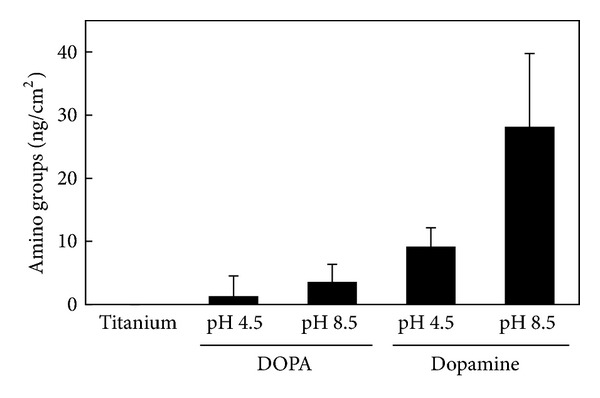
Amounts of amino groups on DOPA- and dopamine-treated titanium.

The amount of amino groups present on DOPA- or dopamine-treated surfaces was measured using FITC (Figure 3). The amount of amino groups in the organic layer was about 3-fold greater at pH 8.5 than pH 4.5 in both treatments; the increase was greater than that of thickness. On the other hand, more amino groups were formed by dopamine than DOPA. Because the amount of amino groups did not increase linearly with increasing thickness, the carboxyl group in DOPA reacted with amino group and reduced it.

**Figure 4:**
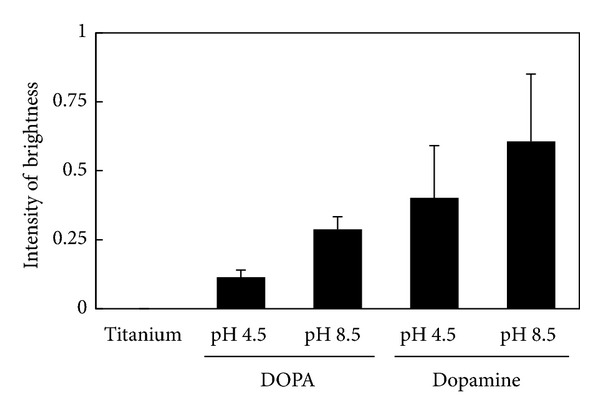
Amounts of BMP2 immobilized on DOPA- and dopamine-treated titanium.

**Figure 5:**
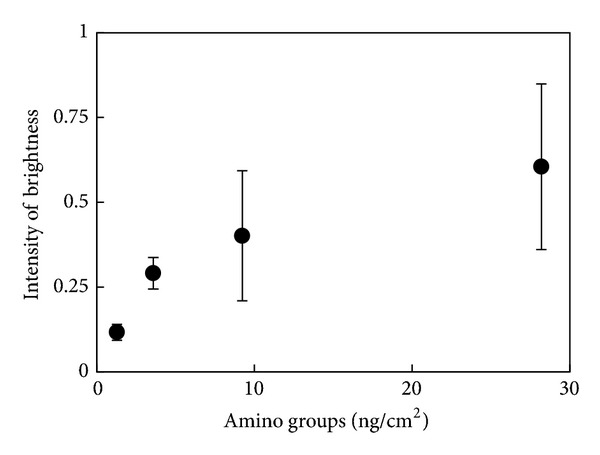
The relationship between the amino groups (from Figure 3) and immobilized BMP2 (from Figure 4).

BMP2 was immobilized by WSC on both the DOPA- and dopamine-treated titanium surfaces, and the immobilization was confirmed by anti-BMP2 antibody (Figure 4). The surface was treated with the BMP2 solution in the absence of WSC and subsequently washed until no nonspecific BMP2 adsorption was detected by anti-BMP2 antibody. The same washing condition was employed for the surfaces treated in the presence of WSC. More BMP2 was immobilized on the surface treated with dopamine at pH 8.5 than pH 4.5. In addition, there was more BMP2 immobilized on the dopamine-treated surfaces than the DOPA-treated surfaces. BMP2 immobilization increased monotonously with increasing amino groups in the layer as shown in Figure 5.

### 3.2. Biological Activity

**Figure 6:**
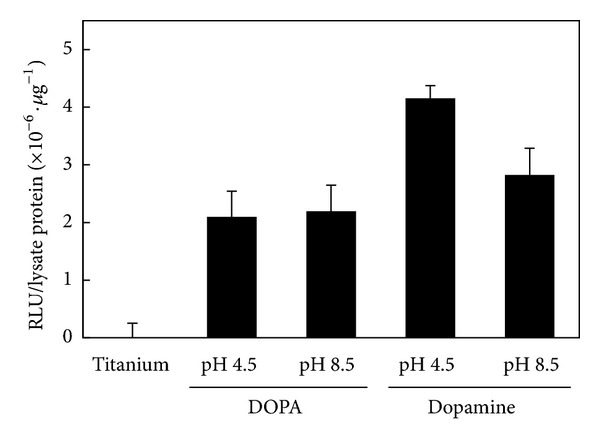
Induction of luciferase activity in BRE-Luc C2C12 cells by immobilized BMP2 on titanium.

After confirming the immobilization of BMP2 on titanium surfaces, BRE-Luc C2C12 cells were seeded and incubated for 2 days. Id proteins act as dominant-negative inhibitors of basic helix-loop-helix transcription factors. Id and basic helix-loop-helix proteins dictate cellular programs of differentiation and proliferation in various cell types in an opposing manner. BMP2 inhibits myogenic differentiation and regulates bone formation. Id1 is strongly induced by BMP2 and is an important mediator of the inhibitory effect of BMP2 on myogenic differentiation [36, 37]. The results of luciferase activity indicate that BMP2 immobilized on titanium surfaces significantly activated the reporter gene (Figure 6). Thus, the results demonstrate that BMP2 interacts with the receptor even after immobilization. The induction on dopamine-treated and BMP2-immobilized surfaces was more enhanced than on DOPA-treated and BMP-immobilized surfaces, although the difference was not significant.

**Figure 7:**
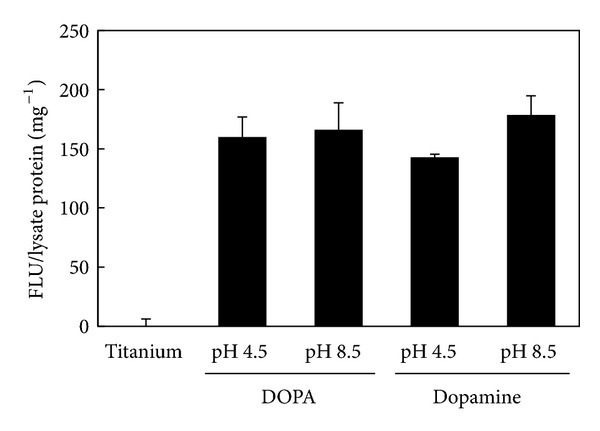
Induction of alkaline phosphatase activity in BRE-Luc C2C12 cells by immobilized BMP2 on titanium.

An alkali phosphatase assay was performed on C2C12 cells cultured for 10 days on the titanium surface with immobilized BMP2 (Figure 7). These cells are usually employed to study the differentiation of myoblasts and osteoblasts. Alkaline phosphatase is a marker of bone formation; its induction indicates cell differentiation from the myoblastic to the osteoblastic lineage. Although no significant difference of cultured cells was observed by microscopy, the immobilized BMP2 induced osteogenic differentiation. There was no significant difference between BMP2-immobilized surfaces.

Covalent immobilization using functional groups in BMP is categorized into amino groups and carboxyl groups. Tsujigiwa et al. [9], Park et al. [11], Schmoekel et al. [12], and Lai et al. [35] used amino groups in BMP. On the other hand, Puleo et al. [21] used carboxyl groups in BMP. In this study, we employed carboxyl groups in BMP for covalent immobilization and found a significant effect of immobilized BMP.

## 4. Conclusions

BMP2 is covalently immobilized on dopamine-treated titanium surfaces. The immobilized BMP2 specifically interacts with myoblasts and induces osteogenic differentiation. Therefore, the present method is convenient for covalently immobilizing BMP2 while retaining its biological activity.

## Figures and Tables

**Table 1:** Water contact angle and thickness of the DOPA and dopamine layers formed on titanium.

Treatment	pH	Water contact angle (°)	Thickness (nm)
—	—	0	0

DOPA	4.5	64.0 ± 1.9	0.676 ± 0.017
	8.5	67.8 ± 1.4	3.225 ± 0.073

Dopamine	4.5	51.4 ± 1.9	0.798 ± 0.073
	8.5	54.7 ± 1.1	22.05 ± 1.048
